# Letter to the editor: Further identification of a measles variant displaying mutations impacting molecular diagnostics, Northern Italy, 2024

**DOI:** 10.2807/1560-7917.ES.2023.29.7.2400079

**Published:** 2024-02-15

**Authors:** Clara Fappani, Maria Gori, Silvia Bianchi, Marta Canuti, Daniela Colzani, Melissa Baggieri, Silvia Gioacchini, Emilio D’Ugo, Elisabetta Tanzi, Fabio Magurano, Antonella Amendola

**Affiliations:** 1 Department of Health Sciences, Università degli Studi di Milano, Milan, Italy; 2 Department of Clinical Sciences and Community Health, Università degli Studi di Milano, Milan, Italy; 3 Coordinated Research Center “EpiSoMI”, Università Degli Studi di Milano, Milan, Italy; 4 These authors contributed equally to this work and share first authorship.; 5 Department of Veterinary and Animal Sciences, University of Copenhagen, Frederiksberg, Denmark; 6 Department of Infectious Diseases, Istituto Superiore di Sanità, Rome, Italy

**Keywords:** Measles, Variants, Surveillance, Molecular methods, Italy

**To the editor**: We read with great interest the article by Pérez-Rodríguez et al. [1], reporting the circulation of measles virus (MeV) variants (genotype D8) displaying three mutations impacting molecular diagnostics. The mutations occurred in the 450 nt-long C-terminal (N-450) region of the nucleoprotein (N) gene that is usually the target of real-time reverse-transcription (RT)-PCR assays commonly employed by surveillance laboratories. In detail, the three T-to-C synonymous substitutions were located within the annealing site of the reverse primer recommended by the United States Centers for Diseases Control and Prevention (CDC, Atlanta) [2]. This results in a slight loss of test sensitivity.

As a Subnational Reference Laboratory of the measles and rubella surveillance network MoRoNet [3], we currently employ a real-time RT-PCR assay using the primers and probe described by Hübschen et al. [4], to amplify a fragment of 114 nt of the N gene that does not fall within the N-450 region. Since January 2024, we have confirmed five measles cases in the surveyed area, which includes the Metropolitan City of Milan and surrounding areas in Lombardy, Northern Italy (ca 4 million inhabitants). This region borders Switzerland, where the study by Pérez-Rodríguez and colleagues was conducted. The five Lombard cases were all classified as genotype D8 and determined to be sporadic since we found no clear epidemiological link among them. In three of these cases, a recent history of travel was reported, and destinations included Uzbekistan, Thailand, and Southern Italy. In particular, two of these cases, each with a travel history to either Southern Italy or Thailand, were infected with MeV strains characterised by the three mismatches described by Pérez-Rodríguez et al. [1].

Both cases were confirmed through the detection of virus-specific IgM in serum with an enzyme-linked immunosorbent assay, and of MeV-RNA in both urine and oropharyngeal swab samples by real-time RT-PCR [4]. MeV strains were genotyped by sequencing the N-450 region as recommended by the World Health Organization (WHO) [5,6]. Both sequences were annotated and deposited in the WHO Measles Virus Nt Surveillance (MeaNS2) database [7] with the distinct sequence identifier (DSId) 8491, and in GenBank [8] (accession numbers: PP334141–PP334142). Notably, the DSId was different from the one (i.e. 8248) detected by Pérez-Rodríguez et al. [1]. Basic Local Alignment Search Tool (BLAST) analysis showed that they shared 99.7% identity with strains identified in Moscow (Russia) in 2023 (GenBank accession numbers: OR290099–OR290102, OR840959, OR840960) [9].

None of the other 614 strains (453 D8 and 161 B3) detected by our laboratory between 2017 (the beginning of surveillance activities) and 2023 possessed these mutations. However, our results suggest that MeV with the specific mutations detected through the Swiss molecular surveillance are already circulating in Italy, in line with the results obtained by Pérez-Rodríguez et al., who reported a local case with travel history to Italy.

We commend Pérez-Rodríguez and colleagues for the rapid communication as they raise awareness on the circulation of a MeV variant that can be detected with reduced sensitivity by many currently used diagnostic tests. This letter confirms their finding and reports that the identified variant is spreading. This highlights the importance of promptly updating diagnostic tests to detect all currently circulating MeV strains.
